# Comparison of atrial arrhythmias and autonomic function parameters according to atrial septal defect closure techniques

**DOI:** 10.3389/fcvm.2026.1737612

**Published:** 2026-01-26

**Authors:** Akif Kavgacı, Semiha Terlemez, Sercan Tak, Fatma İncedere, Lazgin Tuncar, Gizem Karkın Tozlu, Fatma Hayvacı Canbeyli, Serdar Kula, Erkan İriz, Ayşe Deniz Oğuz, Sedef Tunaoğlu

**Affiliations:** 1 Department of Pediatric Cardiology, Faculty of Medicine, Gazi University, Ankara, Türkiye; 2 Department of Pediatric Cardiovascular Surgery, Faculty of Medicine, Gazi University, Ankara, Türkiye

**Keywords:** 24 h ambulatory electrocardiogram, heart rate variability, lowns grade, supraventricular premature contraction, surgical ASD closure, transcatheter ASD closure

## Abstract

**Background:**

Atrial septal defects (ASDs) are associated with an increased risk of atrial arrhythmias due to right atrial dilation, electrical remodeling, and conduction abnormalities. In addition to arrhythmias, autonomic dysfunction may also occur. Although several studies have investigated the impact of transcatheter ASD closure on arrhythmia risk in pediatric patients, direct comparative analyses between surgical and transcatheter closure techniques remain limited in the current literature.

**Methods:**

This study included patients who underwent ASD closure via surgical or transcatheter methods before age 18 and had at least 12 months of follow-up. A control group of healthy, age- and sex-matched children without cardiac disease was also included. All participants underwent 12-lead electrocardiography (ECG) and 24 h Holter monitoring. Patient data included arrhythmia symptoms, closure method, age at closure, defect size, and catheterization findings. Individuals with other cardiac anomalies, genetic syndromes, or medications affecting conduction were excluded.

**Results:**

The study included 131 participants: 91 ASD patients (56 surgical, 35 transcatheter) and 40 controls. Supraventricular premature beats (SVPB) was significantly more frequent in both intervention groups compared to controls, with the highest frequency in the surgical group (*p* < 0.001). *P*-wave dispersion was also highest in the surgical group. In the surgical group, Lowns grade correlated positively with Qp/Qs, mean pulmonary artery pressure, and follow-up duration. Heart rate variability (HRV) parameters were significantly lower in the surgical group, indicating sympathetic dominance.

**Conclusion:**

Atrial septal defect repair increases atrial arrhythmia risk, particularly following surgical intervention. While autonomic function remained comparable to controls after transcatheter closure, surgical closure was associated with reduced HRV and increased sympathetic activity.

## Introduction

Atrial septal defect (ASD) is a prevalent congenital heart disease, comprising about 10% of all congenital cardiac anomalies (1). Surgical repair is typically indicated for ostium primum, sinus venosus ASDs, and specific secundum ASDs unsuitable for device closure (2). Recently, the transcatheter approach for secundum ASDs has gained significant popularity due to its advantages, including avoidance of sternotomy and cardiopulmonary bypass, shorter hospital stays, reduced need for blood transfusions, and decreased patient discomfort (3).

Atrial septal defect is linked to a heightened risk of arrhythmias and conduction disorders due to right-sided volume overload and subsequent electrical remodeling (4). Consequently, the risk of arrhythmias escalates with delayed repair, increased shunt size, and additional factors such as pulmonary hypertension and comorbidities (5). Conversely, the incidence of arrhythmias tends to decrease following ASD closure. Sinus node dysfunction is an infrequent complication, particularly associated with older surgical repair techniques (6). Nonetheless, surgical closure of ASD enhances atrioventricular (AV) conduction, reduces the AV nodal refractory period, and improves sinus node function, likely by alleviating right-sided volume overload. However, sinus node function can be compromised as a result of the surgical intervention (7). Nevertheless, studies in the literature indicate that complications such as atrial arrhythmias and reversible or permanent atrioventricular (AV) block may also increase in children following transcatheter ASD closure (8–11).

While only a limited number of studies in the literature have investigated the effects of transcatheter ASD closure on arrhythmias in children, no study to date has compared the impact of surgical vs. transcatheter closure on atrial arrhythmias specifically in the pediatric population. The aim of this study was to compare the long-term effects of transcatheter and surgical ASD repair on arrhythmia frequency, atrial arrhythmia tendency, and autonomic nervous system balance (sympathetic/parasympathetic activity) in children aged 0–18 years.

## Material and methods

### Study population

A total of 131 participants were included: 91 children with atrial septal defect who underwent closure (surgical repair or transcatheter device implantation) at the Pediatric Cardiology Department of Gazi University Faculty of Medicine with ≥12 months elapsed since repair, and 40 age- and sex-matched healthy controls without structural heart disease. For patients, arrhythmia-related symptoms (palpitations, syncope), medication use, follow-up duration, age at closure, cardiac catheterization parameters (pulmonary/systemic flow ratio and mean pulmonary artery pressure), defect size, closure method, device type for transcatheter procedures, and patch use for surgical repair were recorded.

### Electrocardiography and holter monitoring

All participants underwent a standard 12-lead resting electrocardiogram and 24 h ambulatory Holter electrocardiography (CardioNavigator Plus Impresario, Delmar Reynolds, Paris, France). Electrodes were positioned at the right and left mid-clavicular lines and the left anterior axillary line at the fifth rib level. Recordings were reviewed by a single physician; artifacts were excluded, and studies with <23 h of analyzable data were not accepted. On the resting electrocardiogram, the presence of atrioventricular (AV) block, minimum P-wave duration (Pmin), maximum P-wave duration (Pmax), and P-wave dispersion (Pdis) were assessed. P-wave onset was defined as the intersection of the isoelectric baseline with the initial deflection, and termination as the return to the isoelectric line; P-wave dispersion was calculated as the difference between maximum and minimum P-wave durations across the 12 leads. On Holter monitoring, supraventricular premature beats (SVPBs) were classified as unifocal, bifocal, or multifocal, and SVPB burden was expressed as the proportion of total beats; supraventricular tachycardia (SVT) was recorded and categorized as sustained or non-sustained. Ventricular premature beats (VPBs) were evaluated for morphology, ventricular tachycardia (VT) was identified and categorized as sustained or non-sustained, and VPB burden was expressed as the proportion of total beats. Ventricular arrhythmias were graded according to the Lown classification (grades 0–5) (12). Time-domain heart rate variability (HRV) analysis included mean, maximum, and minimum heart rates, and the following prespecified indices: the standard deviation of all the normal RR intervals (SDNN) (milliseconds), the standard deviation of the means of all the five-minute segment normal RR intervals (SDANN) (milliseconds), the square root of the mean of the sum of the squares of differences between adjacent RR intervals (rMSSD) (milliseconds), the proportion of adjacent RR intervals that differ by more than 50 ms in the 24 h recording (pNN50) (percent), the mean of all the five-minute standard deviations of normal RR intervals during the 24 h period (SDNN index) (milliseconds), and the triangular index.

### Exclusion criteria and consent

Patients were excluded for concomitant cardiac pathology, genetic anomalies, chronic systemic disease, or use of medications affecting cardiac conduction. Controls were excluded if electrocardiogram or Holter findings suggested genetic or acquired arrhythmogenic disorders (e.g., pre-excitation, documented VT or SVT). Written informed consent was obtained from parents of all participating children.

### Statistical analysis

Descriptive statistics were presented as the median and interquartile range values for continuous variables, and as frequencies and percentages for categorical variables. The distribution of continuous variables was evaluated using the Shapiro–Wilk test in conjunction with visual inspection of histograms and Q–Q plots. Multivariable regression analyses were performed using multiple linear regression for continuous outcomes and logistic regression for dichotomous outcomes. Independent group comparisons were conducted using Chi-square tests for categorical variables and the Kruskal–Wallis test for non-normally distributed and/or ordinal variables. The Mann–Whitney U-test was performed to test the significance of pairwise differences, with Bonferroni correction applied to adjust for multiple comparisons. Associations between non-normally distributed and/or ordinal variables were assessed using correlation coefficients, with their significance calculated by the Spearman test. All statistical analyses were performed using SPSS, version 25.0 (IBM Inc., Armonk, NY, USA), and a type I error rate of 5% (*p* < 0.05) was considered the threshold for statistical significance.

All methods were carried out in accordance with relevant guidelines and regulations. This study was conducted in compliance with the principles of the Declaration of Helsinki and was approved by the Ethics Committee of Gazi University.

## Results

A total of 131 children were included in the study, of whom 91 were diagnosed with atrial septal defect (ASD), while 40 children were included as healthy controls. Among the 91 patients with ASD, 35 underwent defect closure via transcatheter approach, and 56 had their defect closed surgically. The demographic data of the patients and healthy controls included in the study are presented in Table 1.

**Table 1:** The demographic data of the patients and healthy controls.

Variable	Transcatheter^a^ (*n* = 35)	Surgical^b^ (*n* = 56)	Healthy control^c^ (*n* = 40)	*p*	p ^(a−b)^	p ^(a−c)^	p ^(b−c)^
Gender, *n* %				0.522			
Male	14 (40%)	28 (50%)	16 (40%)				
Female	21 (%60)	28 (50%)	24 (60%)				
Weight (kg)	54 (15–84)	36 (18–92)	43.5 (15–93)	0.015	0.011	0.182	0.045
Height (cm)	158 (96–179)	143 (105–184)	154 (98–173)	0.005	0.002	0.154	0.060
Age (year)	16 (3.5–27)	11.3 (4–18)	13.8 (3.5–17.5)	0.001	<0.001	0.038	0.066
Closure Age (year)	8 (2.5–17)	6 (1–15)			<0.001		
Follow-up duration (months)	50 (13–225)	49 (14–160)			0.613		

* A *p*-value below 0.017 is considered statistically significant in accordance with the Bonferroni correction.

All patients included in the study were evaluated using a 12-lead spot electrocardiogram (ECG). None of the healthy controls exhibited AV block on the 12-lead ECG, while first-degree AV block was detected in 2.9% of the patients who underwent transcatheter ASD closure and in 8.9% of the patients who underwent surgical ASD closure. No second- or third-degree AV block was observed in any of the patients or healthy controls during electrocardiographic examination.

P-wave indices differed among groups. Pmin was higher in both the transcatheter closure group (median 82 ms) and the surgical closure group (median 81 ms) than in healthy controls (median 68 ms; *p* = 0.004 and *p* = 0.001, respectively), with no difference between the two patient groups (*p* = 0.781). Pmax was greatest in the surgical group (median 148 ms) vs. the transcatheter group (median 134 ms; *p* = 0.001) and controls (median 121 ms; *p* < 0.001). Pdis was likewise highest in the surgical group (median 63 ms) compared with the transcatheter group (median 42 ms; *p* < 0.001) and controls (median 47 ms; *p* < 0.001), while the transcatheter group and controls did not differ (*p* = 0.591).The electrocardiogram data for Pmin, P max, and P dis values, as well as the correlation analysis between them, are presented in Table 2.

**Table 2:** Analysis of P Minimum, P Maximum, and P dispersion values on electrocardiogram across groups and their correlation.

Parameter	Transcatheter^a^	Surgical^b^	Healthy control^c^	*p*	p ^(a−b)^	p ^(a−c)^	p ^(b−c)^
	Median (25–75p)	Median (25–75p)	Median (25–75p)				
Pmin (msn)	82 (70–105)	81 (74–93)	68 (63–83)	0.002	0.781	0.004	0.001
P max (msn)	134 (120–142)	148 (132–168)	121 (110–136)	<0.001	0.001	0.038	<0.001
Pdis (msn)	42 (30–60)	63 (50–78)	47 (37–56)	<0.001	<0.001	0.591	<0.001

Pmin, minimum P-wave duration; Pmax, maximum P-wave duration; Pdis, P-wave dispersion.

* A *p*-value below 0.017 is considered statistically significant in accordance with the Bonferroni correction.

In multivariable linear regression analysis, Qp/Qs ratio and closure technique were independently associated with P-wave dispersion. Age at closure (B = 0.582, 95% CI: −0.548 to 1.712, *p* = 0.309) and mean pulmonary artery pressure (B = 0.182, 95% CI: −0.799 to 1.163, *p* = 0.714) were not independently associated with P-wave dispersion and were therefore excluded from the final model. Increasing Qp/Qs ratio was a strong predictor of higher P-wave dispersion (B = 24.220, 95% CI: 15.461–32.978, *p* < 0.001). Closure technique also remained independently associated with P-wave dispersion; compared with transcatheter closure, surgical closure was associated with a significantly higher P-wave dispersion (B = 11.228, 95% CI: 2.742–19.714, *p* = 0.010**)** (Table 3).

**Table 3:** Multivariable linear regression analyses.

Parameter	Parameter	B	95% CI	*p* value
Pdis (msn)	Qp/Qs ratio	24.220	15.461 to 32.978	<0.001
	Closure technique	11.228	2.742 to 19.714	0.010
SDNN	Age at closure	1.849	0.133 to 3.565	0.035
	Qp/Qs ratio	−17.157	−30.915 to −3.399	0.015
	Closure technique	−75.127	−89.084 to −61.170	<0.001
Lown's grade	Age at closure	−0.084	−0.153 to −0.015	0.017
	Qp/Qs ratio	1.195	0.643 to 1.747	<0.001
	Closure technique	1.402	0.842 to 1.962	<0.001
SVPB frequency (%)	Qp/Qs ratio	2.513	1.508 to 3.518	<0.001
	Closure technique	1.270	0.297 to 2.244	0.011

B, unstandardized regression coefficient; CI, confidence interval, SDNN, the standard deviation of all the normal RR intervals; Qp/Qs, pulmonary-to-systemic blood flow ratio; mPAP, mean pulmonary artery pressure; Pmin, minimum P-wave duration; Pmax, maximum P-wave duration; Pdis, P-wave dispersion; SVPB, supraventricular premature beats.

Heart rate variability indices demonstrated significant differences among the three study groups. Relative to healthy controls, the transcatheter cohort largely preserved variability, whereas the surgical cohort exhibited globally reduced values. In two-group comparisons, transcatheter vs. surgical differences were significant for SDNN, SDNN index, pNN50, rMSSD, SDANN, and the triangular index (all *p* < 0.001). Transcatheter vs. control comparisons showed a higher SDNN (*p* = 0.002) with no differences for SDNN index, pNN50, rMSSD, or the triangular index; the SDANN difference (*p* = 0.042) did not meet the multiplicity-adjusted threshold. Surgical vs. control comparisons were significant for all indices. Detailed results, together with the correlation analyses, are provided in Table 4.

**Table 4:** Analysis of heart rate variability values on 24 h ambulatory electrocardiogram across groups and their correlation.

Parameter	Transcatheter^a^	Surgical^b^	Healthy control^c^	*p*	p ^(a−b)^	p ^(a−c)^	p ^(b−c)^
	Median (25–75p)	Median (25–75p)	Median (25–75p)				
SDNN	195 (168–212)	94 (82–113)	162 (137–182)	<0.001	<0.001	0.002	<0.001
SDNN ıdx	103 (95–160)	57 (48–78)	102 (95–118)	<0.001	<0.001	0.226	<0.001
pNN50 (%)	36 (25–42)	12 (11–18)	31 (26–35)	<0.001	<0.001	0.244	<0.001
rMSSD	65 (54–78)	27 (18–35)	57 (53–73)	<0.001	<0.001	0.470	<0.001
SDANN	144 (126–178)	85 (72–110)	129 (113–149)	<0.001	<0.001	0.042	<0.001
Triangular index	38 (30–44)	19 (14–25)	39 (32–44)	<0.001	<0.001	0.614	<0.001

pNN50%, the proportion of adjacent RR intervals that differ by more than 50 ms in the 24 h recording; RMSSD, the square root of the mean of the sum of the squares of differences between adjacent RR intervals; SDANN, the standard deviation of the means of all the five-minute segment normal RR intervals; SDNN, the standard deviation of all the normal RR intervals; SDNN index, the mean of all the five-minute standard deviations of normal RR intervals during the 24 h period.

* A *p*-value below 0.017 is considered statistically significant in accordance with the Bonferroni correction.

In multivariable linear regression analysis, age at closure, Qp/Qs ratio, and closure technique were independently associated with SDNN. Mean pulmonary artery pressure was included in the initial multivariable model but was not independently associated with SDNN (B = 0.428, 95% CI: −1.104 to 1.959, *p* = 0.580) and was therefore excluded from the final model. Older age at closure was associated with higher SDNN values (B = 1.849, 95% CI: 0.133–3.565, *p* = 0.035), whereas increasing Qp/Qs ratio was associated with lower SDNN (B = −17.157, 95% CI: −30.915 to −3.399, *p* = 0.015). Closure technique showed the strongest independent association with SDNN; compared with transcatheter closure, surgical closure was associated with a markedly lower SDNN (B = −75.127, 95% CI: −89.084 to −61.170, *p* < 0.001) (Table 3).

In the analysis of 24-hour ambulatory ECG findings, significant differences were observed among the three groups across multiple parameters. Supraventricular premature beats (SVPB) distribution differed across cohorts, with unifocal morphology predominating after transcatheter closure, whereas bifocal and multifocal forms were overrepresented after surgical closure (global *p* < 0.001). Two-group comparisons showed a higher frequency of SVPB couplets in the surgical cohort than in both the transcatheter cohort and controls (*p* < 0.001). Non-sustained SVT was more frequent after surgery than after transcatheter closure and vs. controls (*p* = 0.008 and *p* = 0.004), while sustained SVT did not differ between groups. Nodal premature beats were likewise more prevalent after surgery (*p* = 0.014 vs. transcatheter; *p* = 0.005 vs. controls). Rare VES were present in 25.7% of transcatheter and 38.5% of surgical ASD closure patients, but absent in healthy controls (*p* = 0.001 for transcatheter vs. control, *p* < 0.001 for surgical vs. control). Lowns grades were shifted toward higher categories in the surgical cohort compared with both comparators (*p* < 0.001). Detailed distributions, including subgroup analyses of patients and healthy controls, are presented in Table 5.

**Table 5:** Findings from 24 hour ambulatory ECG by group and subgroup analysis between groups.

Parameter		Transcatheter^a^		Surgical^b^		Healthy control^c^		*p*	*p* ^(a−b)^	*p* ^(a−c)^	*p* ^(b−c)^
		n	%	n	%	n	%				
SVPB	None	0	0	0	0	12	30	<0.001	<0.001	<0.001	<0.001
	Unifocal	28	80	9	16.1	28	70				
	Bifocal	7	20	29	51.8	0	0				
	Multifocal	0	0	18	32.1	0	0				
Couplet SVPB	None	26	74.3	6	10.7	40	100	<0.001	<0.001	0.001	<0.001
	Present	9	25.7	50	89.3	0	0				
SVT (non-sustained)	None	33	94.3	40	71.4	38	95	0.001	0.008	0.891	0.004
	Present	2	5.7	16	28.6	2	5				
SVT (sustained)	None	35	100	52	92.9	40	100	0.063	0.106	-	0.084
	Present	0	0	4	7.1	0	0				
Nodal premature beat	None	34	97.1	41	73.2	33	82.5	<0.001	0.014	0.058	0.005
	Rare	0	0	2	3.6	6	15				
	Present	1	2.9	13	23.2	1	2.5				
VES	None	26	74.3	31	59.6	40	100	<0.001	0.304	0.001	<0.001
	Rare	9	25.7	21	40.4	0	0				
Lowns grade	0	0	0	0	0	12	30	<0.001	<0.001	0.083	<0.001
	1	21	60	3	5.4	10	25				
	2	7	20	9	16.1	18	45				
	3	1	2.9	2	3.6	0	0				
	4	3	8.6	25	44.6	0	0				
	5	3	8.6	12	21.4	0	0				
	6	0	0	5	8.9	0	0				
SVPB frequency % (Median (25–75p)		0.50 (0.10–1)		2.00 (0.90–4.50)		0.10 (0–0.60)		<0.001	<0.001	0.006	<0.001

SVPB, supraventricular premature beat; SVT, supraventricular tachycardia; VES, ventricular extrasystoles.

* A *p*-value below 0.017 is considered statistically significant in accordance with the Bonferroni correction.

In multivariable linear regression analysis, age at closure, Qp/Qs ratio, and closure technique were independently associated with Lown's grade. Mean pulmonary artery pressure was not independently associated with Lown's grade (B = 0.034, 95% CI: −0.027 to 0.095, *p* = 0.276) and was therefore excluded from the final model. Younger age at closure was associated with higher Lown's grade (B = −0.084, *p* = 0.017). Increasing Qp/Qs ratio was also an independent predictor of higher Lown's grade (B = 1.195, *p* < 0.001). Closure technique remained independently associated with Lown's grade; compared with transcatheter closure, surgical closure was associated with a significantly higher Lown's grade (B = 1.402, 95% CI: 0.842–1.962, *p* < 0.001). In multivariable linear regression analysis, Qp/Qs ratio and closure technique were independently associated with SVPB frequency. Age at closure (B = −0.054, 95% CI: −0.182 to 0.075, *p* = 0.408) and mean pulmonary artery pressure (B = 0.076, 95% CI: −0.035 to 0.187, *p* = 0.176) were not independently associated with SVPB frequency and were therefore excluded from the final model. Increasing Qp/Qs ratio was a strong predictor of higher SVPB frequency (B = 2.513, 95% CI: 1.508–3.518, *p* < 0.001). Closure technique also remained independently associated with SVPB frequency; compared with transcatheter closure, surgical closure was associated with a significantly higher SVPB frequency (B = 1.270, 95% CI: 0.297–2.244, *p* = 0.011) (Table 3).

In correlation analyses integrating 24-hour ambulatory ECG indices with clinical variables, several robust and additional significant associations were identified. In the transcatheter group, Pmax was significantly correlated with age (*p* < 0.001) and SDNN was significantly correlated with SDANN (*p* < 0.001); HRV indices (pNN50, rMSSD, triangular index) were inversely related to Lown's grade, with several associations reaching *p* < 0.001 (overall range *p* < 0.001–0.014). In the surgical cohort, Pmin was significantly correlated with Pmax (*p* < 0.001), and Pmax correlated not only with Pdis (*p* < 0.001) but also with Lown's grade (*p* = 0.004) and surgical closure method (*p* = 0.030). The SDNN index was significantly correlated with SDNN (*p* < 0.001) and with surgical method (*p* = 0.010), while showing an inverse association with Lown's grade (*p* < 0.001). Similarly, the triangular index correlated positively with surgical technique (*p* = 0.012) and negatively with Lown's grade (*p* < 0.001), indicating that procedural approach and patient-specific factors are linked to variation in atrial conduction and time-domain HRV metrics in the surgical ASD group. In the healthy control cohort, Pmin was positively correlated with Pmax (*p* < 0.001) and inversely correlated with Pdis (*p* = 0.047). Pmax was positively correlated with Pdis (*p* = 0.001). In addition, the SDNN index was positively correlated with the triangular index (*p* = 0.023). These associations indicate coherent interrelations among atrial conduction measures and time-domain HRV metrics even in healthy children. The correlation analysis of 24-hour ambulatory ECG data with various parameters across the three study groups is detailed in Table 6.

**Table 6:** Correlation of 24 h ambulatory ECG data with demographics, catheter angiography findings, echocardiography data, device size, and follow-up duration.

Parameter	Transcatheter			Surgical			Healthy control		
	Parameter	*p*	Correlation coefficient	Parameter	*p*	Correlation coefficient	Parameter	*p*	Correlation coefficient
Pmin (msn)	Lowns grade	0.046	−0.340	Pmax (msn)	<0.001	0.553	Pmax (msn)	<0.001	0.565
	Pmax (msn)	0.001	0.550	Weight	0.017	0.319	Pdis (msn)	0.047	−0.317
	Pdis (msn)	0.008	−0.441	Height	0.027	0.296			
	SDNN ıdx	0.015	0.408						
	Weight	0.008	0.443						
	Height	<0.001	0.646						
	Age (year)	<0.001	0.672						
	Closure age (year)	0.029	0.369						
Pmax (msn)	Pmin (msn)	0.001	0.550	Surgical closure method	0.03	−0.290	Pmin (msn)	<0.001	0.565
	Pdis(msn)	0.012	0.420	Lowns grade	0.004	0.376	Pdis (msn)	0.001	0.495
	Height	0.006	0.455	Pmin (msn)	<0.001	0.553			
	Age (year)	<0.001	0.559	Pdis (msn)	<0.001	0.774			
	Closure age (year)	<0.001	0.565	pNN50 (%)	0.017	−0.318			
				Weight	0.001	0.435			
				Height	0.002	0.402			
				Age (year)	0.006	0. 361			
				Qp/Qs	<0.001	0.540			
				Largest diameter of defect	0.018	0.316			
				Follow-up duration (month)	0.045	0.269			
Pdis (msn)	Pmin (msn)	0.008	−0,441	Surgical closure method	0.001	−0.432	Pmin (msn)	0.047	−0.317
	Pmax(msn)	0.012	0.420	Lowns grade	<0.001	0.553	Pmax (msn)	0.001	0.495
				P max (msn)	<0.001	0.774			
				pNN50 (%)	<0.001	−0,468			
				rMSSD	0.002	−0.399			
				Qp/Qs	<0.001	0.582			
				Follow-up duration (month)	0.042	0.273			
SDNN	Device body diameter	0.007	−0.505	Surgical closure method	0.002	0.414			
	SDNN ıdx	<0.001	0.698	Lowns grade	<0.001	−0.633			
	SDANN	<0.001	0.705	SDNN ıdx	<0.001	0.752			
	Height	0.012	0.422	pNN50 (%)	0.001	0.433			
	Age (year)	0.015	0.406	rMSSD	<0.001	0.560			
	Closure age (year)	0.029	0.369	SDANN	<0.001	0.802			
				Triangular index	<0.001	0.467			
				Qp/Qs	0.009	−0.345			
				Closure age (year)	0.043	0.272			
SDNN ıdx	Device body diameter	0.048	−0,383	Surgical closure method	0.01	0.340	Triangular index	0.023	0.358
	Lowns grade	0.023	−0,383	Lowns grade	<0.001	−0.561			
	Pmin (msn)	0.015	0.408	SDNN	<0.001	0.752			
	SDNN	<0.001	0.698	pNN50 (%)	0.009	0.346			
	rMSSD	0.047	0.338	rMSSD	0.006	0.363			
	SDANN	0.023	0.383	SDANN	<0.001	0.701			
	Weight	0.026	0.376	Triangular index	<0.001	0.459			
	Height	0.003	0.488	Qp/Qs	0.02	−0.310			
	Age (year)	0.002	0.503						
	mPAP	0.026	0.377						
	Closure age (year)	0.022	0.385						
	Largest diameter of defect	0.028	−0.371						
pNN50 (%)	Lowns grade	<0.001	−0,581	Surgical closure method	0.001	0.418			
				Lowns grade	<0.001	−0.704			
	rMSSD	0.002	0.502	Pmax (msn)	0.017	−0.318			
	Triangular index	0.014	0,412	Pdis (msn)	<0.001	−0,468			
				SDNN	0.001	0.433			
				SDNN ıdx	0.009	0.346			
				rMSSD	<0.001	0.506			
				SDANN	0.002	0.311			
				Triangular index	0.009	0.345			
				Qp/Qs	0.001	−0.420			
				Largest diameter of defect	0.012	−0,334			
rMSSD	Lowns grade	<0.001	−0.626	Surgical closure method	<0.001	0.452			
				Lowns grade	<0.001	−0.676			
	SDNN ıdx	0.047	0.338	Pdis(msn)	0.002	−0.399			
	pNN50 (%)	0.002	0,502	SDNN	<0.001	0.560			
				SDNN ıdx	0.006	0.363			
				pNN50 (%)	<0.001	0.506			
				SDANN	<0.001	0.454			
				Triangular index	0.003	0.385			
				Qp/Qs	<0.001	−0.475			
				mPAP	0.021	−0.308			
				Largest diameter of defect	0.014	−0.328			
SDANN	SDNN	<0.001	0.705	Surgical closure method	0.001	0.415			
	Device body diameter	0.026	−0,427	Lowns grade	<0.001	−0.560			
	Lowns grade	0.011	−0,423	SDNN	<0.001	0.802			
	SDNN ıdx	0.023	0.383	SDNN Idx	<0.001	0.701			
				pNN50 (%)	0.002	0.311			
				rMSSD	<0.001	0.454			
				Triangular index	<0.001	0.502			
				Qp/Qs	0.007	−0.358			
Triangular index	pNN50 (%)	0.014	0,412	Surgical closure method	0.012	0.335	SDNN ıdx	0.023	0.358
				Lowns grade	<0.001	−0.498			
				SDNN	<0.001	0.467			
				SDNN Idx	<0.001	0.459			
				pNN50 (%)	0.009	0.345			
				rMSSD	0.003	0.385			
				SDANN	<0.001	0.502			

pNN50%, the proportion of adjacent RR intervals that differ by more than 50 ms in the 24 h recording; RMSSD, the square root of the mean of the sum of the squares of differences between adjacent RR intervals; SDANN, the standard deviation of the means of all the five-minute segment normal RR intervals; SDNN, the standard deviation of all the normal RR intervals; SDNN index, the mean of all the five-minute standard deviations of normal RR intervals during the 24 h period; Qp/Qs, pulmonary-to-systemic blood flow ratio; mPAP, mean pulmonary artery pressure; Pmin, minimum P-wave duration; Pmax, maximum P-wave duration; Pdis, P-wave dispersion.

## Discussion

Atrial septal defects are common congenital cardiac anomalies characterized by deficiency or absence of interatrial septal tissue. Small defects may close spontaneously, whereas larger defects typically require transcatheter or surgical intervention; if unrepaired, they may lead to right-sided volume overload, atrial arrhythmias, and pulmonary arterial hypertension (13, 14). Resultant right-sided volume loading and concomitant electrical remodeling increase the risk of arrhythmias and conduction disturbances, contributing to the higher prevalence of atrial tachyarrhythmias in this population (4). In line with these pathophysiologic alterations, electrocardiographic patterns are considered to reflect atrial structural remodeling, predominantly right atrial enlargement, whereas right-axis deviation and incomplete right bundle-branch block are viewed as secondary to right ventricular pressure and volume overload (15). In routine clinical practice, a standard 12-lead electrocardiogram commonly shows alterations in P-wave morphology, PR-interval prolongation, right-axis deviation, and incomplete right bundle-branch block (iRBBB) (16, 17).

In our study, first-degree AV block was detected in 2.9% of patients who underwent transcatheter closure of ASD, compared to 8.9% in those who underwent surgical closure. In the transcatheter ASD closure group, iRBBB was observed in 5 patients, while in the surgical group, it was detected in 18 patients. Considering that patients who undergo surgical closure of ASD generally have larger and less suitable defects for transcatheter closure, it is natural that the ECG changes reflecting the morphological alterations caused by the atrial septal defect are observed more frequently in the surgical group in our study. In addition to these findings, our data demonstrated that Pmax values were significantly higher in the surgical group compared to the transcatheter group (*p* = 0.001) and healthy controls (*p* < 0.001). Similarly, Pdis values were found to be significantly higher in the surgical group than in both the transcatheter group and healthy controls (*p* < 0.001). The observation that the surgical group exhibited higher Pmax and Pdis values compared to both the transcatheter group and healthy controls, and that the transcatheter group also had higher values than healthy controls, underscores the impact of atrial morphological alterations caused by ASD. As previously mentioned, the fact that patients in the surgical group predominantly had larger ASDs suggests that these morphological changes are more pronounced in this cohort, thereby rendering the findings particularly significant.

Consistent with the surface electrocardiography findings, ambulatory monitoring demonstrated a greater supraventricular arrhythmic burden in the surgical cohort: supraventricular couplets and supraventricular premature beats were more frequent than in the transcatheter cohort and controls, and non-sustained supraventricular tachycardia and nodal premature beats were likewise increased; frequent ventricular extrasystoles were not observed. Lowns grades were shifted toward higher categories after surgery. Correlation analyses indicated that, in the transcatheter cohort, higher Lowns grades were associated with younger age at closure and larger defects, whereas in the surgical cohort higher grades correlated with greater shunt (Qp/Qs), higher mean pulmonary artery pressure, and longer follow-up, and were inversely related to surgical technique. These findings are consistent with recent literature suggesting an increased long-term risk of atrial arrhythmias following surgical closure. In a meta-analysis conducted by De Liyis and colleagues, including 14 studies and 9,695 pediatric patients, it was reported that the incidence of arrhythmias was significantly higher in those who underwent surgical closure compared to those treated with transcatheter approaches (18). This emphasizes the critical need for structured long-term rhythm follow-up in patients after ASD repair, particularly following surgical closure but also including transcatheter closure, to ensure timely detection and management of arrhythmogenic complications.

Heart rate variability is a noninvasive marker of autonomic regulation and is reported to be reduced in children with ASD, with inverse associations to right-sided filling pressures, supporting a link between volume overload and autonomic dysregulation (19–21). Prior work also suggests that defect closure may partially restore autonomic balance by relieving volume load (21–24). In our cohort, variability was largely preserved after transcatheter closure and approximated age-matched controls, whereas surgical closure was associated with lower SDNN, SDNN index, pNN50, rMSSD, SDANN, and a reduced triangular index. The concordant decrement across indices indicates attenuation of parasympathetic modulation with relative sympathetic predominance, and the persistence of these differences beyond 12 months after repair suggests effects that extend beyond the immediate postoperative period.

In the transcatheter group, Lown's grade was inversely associated with SDNN index and also showed negative correlations with pNN50, rMSSD, and the triangular index, consistent with greater arrhythmic burden in the setting of reduced vagal modulation. In the surgical group, Lown's grade correlated positively with Pmax and Pdis and inversely with the SDNN index; additionally, the SDNN index correlated positively with SDNN and with the surgical method, while the triangular index correlated positively with surgical technique and inversely with Lown's grade. These correlations provide important insight into the mechanisms underlying atrial remodeling and autonomic dysfunction. The positive association between Pmax and P-wave dispersion suggests increasing heterogeneity of atrial conduction with progressive electrical remodeling. Moreover, the association between Pdis and both the pre-closure Qp/Qs ratio and follow-up duration indicates that, particularly in patients with longer follow-up, the severity of pre-procedural volume overload and the timing of repair contribute to persistent atrial conduction abnormalities after surgical intervention. Taken together, these findings suggest that a predisposition to atrial arrhythmias and autonomic dysfunction may persist following surgical closure, whereas the transcatheter group appears largely comparable to age-matched healthy controls in terms of arrhythmic burden and autonomic profile.

In a long-term follow-up study of patients who underwent surgical ASD closure before age 15 (mean postoperative follow-up, 45 years), supraventricular arrhythmias were documented in 69% on Holter monitoring, with short supraventricular tachycardia episodes in 57%, indicating a persistent atrial arrhythmic substrate decades after repair (25). Another study comparing surgically and percutaneously closed ASDs with healthy controls reported significantly reduced HRV across all six parameters (pNN50%, RMSSD, SDANN, SDNN, SDNN index, and triangular index) in the surgical group and lower SDNN, SDANN, and RMSSD in the percutaneous group vs. controls, suggesting that autonomic dysfunction may persist long after closure regardless of technique, albeit less pronounced after percutaneous repair (26).

The absence of pre-procedural 24-hour Holter monitoring in the present study limits the ability to determine whether the observed differences in arrhythmic burden and autonomic function are solely attributable to the closure technique (surgical or transcatheter) itself or partly reflect pre-existing disease severity. Previous studies have shown that children with unrepaired ASD already exhibit increased atrial ectopy, prolonged P-wave indices, and reduced heart rate variability in proportion to shunt magnitude, right atrial enlargement, and pulmonary artery pressure (4, 5, 19, 21). In this context, patients undergoing transcatheter closure may represent a subgroup with smaller or hemodynamically less burdensome defects, which could also allow for intervention at a relatively later age. Accordingly, the higher arrhythmic burden and lower HRV observed in the surgically treated group may, at least in part, represent the persistence of more advanced pre-closure atrial remodeling rather than a pure procedural effect.

Our findings are consistent with prior studies and further suggest that procedural invasiveness also contributes to these differences. Surgical repair, through atriotomy, suture lines, and patch implantation, may promote atrial scarring and conduction heterogeneity, thereby increasing arrhythmogenic susceptibility and impairing autonomic regulation; cardiopulmonary bypass and pericardiotomy may further exacerbate these effects. In contrast, transcatheter closure avoids atrial incisions better preserves atrial geometry, and is associated with a lower arrhythmic burden and a more favorable autonomic profile. Taken together, these observations indicate that long-term rhythm outcomes after ASD closure likely reflect the combined influence of pre-closure disease severity and the invasiveness of the repair technique.

### Limitations

In addition to the limitations posed by the small sample size, we were unable to implement our initial plan of conducting a 24 h ambulatory electrocardiogram study prior to the procedure in patients undergoing surgical or interventional atrial septal defect closure. Therefore, the lack of baseline Holter data limits causal inferences regarding whether post-procedural differences in autonomic function and arrhythmia burden are attributable to the intervention itself or to pre-existing atrial remodeling associated with ASD severity.

## Conclusion

In our study, ASD repair was associated with increased atrial arrhythmic risk, with a substantially greater burden after surgical compared with transcatheter closure, even with follow-up exceeding 12 months. This pattern is consistent with more extensive atrial remodeling following open-heart intervention. Autonomic assessment showed that time-domain heart rate variability in the transcatheter group approximated healthy controls, suggesting preserved autonomic balance, whereas the surgical group exhibited globally reduced indices consistent with sympathetic predominance. Taken together, these observations indicate a potential long-term impact of surgical ASD repair on both arrhythmic susceptibility and autonomic regulation. Prospective, adequately powered, multi-center studies are needed to clarify clinical implications and to optimize post-repair rhythm surveillance strategies.

## Data Availability

The raw data supporting the conclusions of this article will be made available by the authors, without undue reservation.
